# Detecting survival-associated biomarkers from heterogeneous populations

**DOI:** 10.1038/s41598-021-82332-y

**Published:** 2021-02-05

**Authors:** Takumi Saegusa, Zhiwei Zhao, Hongjie Ke, Zhenyao Ye, Zhongying Xu, Shuo Chen, Tianzhou Ma

**Affiliations:** 1 Department of Mathematics, University of Maryland, College Park, MD 20742 USA; 2 Department of Epidemiology and Biostatistics, University of Maryland, College Park, MD 20740 USA; 3 Department of Biostatistics, University of Pittsburgh, Pittsburgh, PA 15213 USA; 4 Division of Biostatistics and Bioinformatics, Department of Epidemiology and Public Health, University of Maryland School of Medicine, Baltimore, MD 21201 USA

**Keywords:** Statistics, Bioinformatics

## Abstract

Detection of prognostic factors associated with patients’ survival outcome helps gain insights into a disease and guide treatment decisions. The rapid advancement of high-throughput technologies has yielded plentiful genomic biomarkers as candidate prognostic factors, but most are of limited use in clinical application. As the price of the technology drops over time, many genomic studies are conducted to explore a common scientific question in different cohorts to identify more reproducible and credible biomarkers. However, new challenges arise from heterogeneity in study populations and designs when jointly analyzing the multiple studies. For example, patients from different cohorts show different demographic characteristics and risk profiles. Existing high-dimensional variable selection methods for survival analysis, however, are restricted to single study analysis. We propose a novel Cox model based two-stage variable selection method called “Cox-TOTEM” to detect survival-associated biomarkers common in multiple genomic studies. Simulations showed our method greatly improved the sensitivity of variable selection as compared to the separate applications of existing methods to each study, especially when the signals are weak or when the studies are heterogeneous. An application of our method to TCGA transcriptomic data identified essential survival associated genes related to the common disease mechanism of five Pan-Gynecologic cancers.

## Introduction

Many biomedical studies aim to understand the progression of a disease and identify prognostic factors of patients’ survival time after standard treatment. Traditional well-known clinical prognostic factors for diseases such as cancer often provide poor prognosis and prediction^1^. The rapid advancement of high-throughput technology in the past two decades has generated enormous amount of genomic data at different levels (e.g. genetic variants, gene expression and DNA methylation) and enabled the tailoring of medical treatment to individual molecular characteristics of each patient^2^. Detection of prognostic genomic biomarkers is useful for the selection of patients who will likely benefit from a specific clinical intervention in precision medicine. Over years, various prognostic genomic biomarkers were reported in the literature but a majority of them are of limited use outside research^3^. As the price of technology becomes more affordable, many genomic studies are conducted to explore a common scientific question in different cohorts or populations. For example, the Pan-Cancer Atlas initiated by The Cancer Genome Atlas (TCGA) consortium studied the multi-platform molecular profiles spanning 33 cancer types and identified common somatic mutations or other genetic variations across multiple tumor lineages^4,5^. Numerous clinical trials were conducted to detect prognostic molecular markers for survival in different cancer types^6–9^. Compared to a single study, discovery of biomarkers from multiple studies is more reproducible and credible and shows stronger evidence of a true association with potential for clinical utility^10^. However, the intrinsic population heterogeneity that exists in different cohorts has created a barrier for an effective integration of multiple studies. For example, high inter-tumor and intra-tumor heterogeneity exhibited in different tumor types present heterogenous patient populations with different risk profiles in Pan-cancer analysis^11,12^. Additional difficulty arises when the time to event outcomes are censored at different time points due to different study durations. Due to these challenges, no methods have been developed to detect survival-associated biomarkers from multiple studies while accounting for the heterogeneity in study populations and designs.

Standard models for the analysis of survival data with censoring include the nonparametric Kaplan–Meier method^13^, the semi-parametric Cox proportional hazards model, abbreviated as the Cox model^14^, and other parametric regression models^15^. Due to the high-dimensional nature of genomic data (i.e., the number of features greatly exceeds the sample size), there is a serious collinearity issue in fitting a prediction model with limited sample size so that these survival models do not directly apply. Classical model selection methods such as the best subset selection using AIC^16^ or BIC^17^ criterion suffer from an NP-hard combinational problem in the presence of a large number of features. There are two major approaches in modern variable selection for high dimensional data. The first approach directly applies popular regularization methods such as Lasso^18^ and elastic net^19^. These regularization methods have been adopted to survival analysis in recent years based on the Cox model^20–22^ or parametric models^23,24^ to detect features associated with the survival outcome. The second approach implements a two-stage procedure by first reducing the dimension of feature space from high to moderate size via sure independence screening (SIS; sure screening or screening for short) methods and then applying regularization methods to refine the final pool of features. First introduced by Fan et al.^25^, SIS comprises a series of methods that select features based on their marginal associations with the response. Zhao et al.^26^ proposed a Cox SIS procedure by fitting marginal Cox models to reduce the dimension in the analysis of censored data. The two-stage procedure has advantages in computational efficiency and algorithmic stability, and has been widely applied in genomic data analysis. However, all the aforementioned variable selection methods are restricted to single study analysis.

In this paper, we propose a novel Cox model based two-stage variable selection method for the detection of survival associated biomarkers common in multiple genomic studies. In the first stage, we extend the screening procedure for the linear model in Ma et al.^27^ to perform sure screening with multiple studies in high-dimensional Cox regression. In the second stage, we penalize the partial log-likelihoods with a group lasso penalty across multiple studies to select the final set of features in all studies simultaneously. The proposed method is statistically attractive in that it allows the studies to have different signal strengths, baseline hazard rates and censoring distributions, and effectively integrates the information from multiple heterogeneous studies. In addition, the method is also computationally favorable by implementing a fast marginal screening in the first stage and solving the regularization problem in the second stage with an efficient ADMM algorithm^28^. To the best of our knowledge, the proposed method is the first to address the high-dimensional variable selection problem in survival models with multiple studies. We also extend other regularization and two-stage methods to a multiple study version to compare our method under various simulation scenarios and demonstrate our method’s strength, especially when the signals are weak or when the studies are heterogeneous. An application of our method in TCGA Gynecologic and breast (Pan-Gynecologic or Pan-Gyn) cancer transcriptomic data detects essential survival associated genes related to the common disease mechanism of Pan-Gyn cancers.

The paper is structured as follows. In Sect. 2, we discuss the variable selection in the Cox model with both single and multiple studies, and introduce our novel two-stage variable selection methodology for multiple studies. Section 3 evaluates the performance of our method under four different simulation scenarios. In Sect. 4, we analyze TCGA Pan-Gyn cancer with the proposed methodology. Discussion and final conclusion are presented in Sect. 5.

## Methods

### Variable selection in Cox model with single study

Suppose there are *n* subjects for right censored survival data. The outcome of the *i*th ($1\le i \le n$) subject is $O_i=(Y_i,\Delta _i)$ where $Y_i$ is the observed survival time and $\Delta _i$ is the censoring indicator. When right censoring occurs, censoring time $C_i$ comes before the true survival time $T_i$. In this case, we only observe $Y_i=C_i$ and $\Delta _i=0$. When the subject is not censored, we observe the true survival time $Y_i=T_i$ with $\Delta _i=1$. The Cox model is a popular semi-parametric survival model to investigate the association between the survival outcome and explanatory variables *X* such as the gene expression through modeling the hazard function by

1 $$\begin{aligned} \lambda (t|X) = \lambda _0(t) \exp (X \beta ), \end{aligned}$$

where $X = (x_1,\ldots , x_p)$ is the feature vector with *p* being the number of features, $\beta = (\beta _1, \ldots , \beta _p)^T$ is the corresponding regression coefficient vector and $\lambda _0(t)$ is the baseline hazard function. In the low-dimensional case where *p* is smaller than *n*, Cox^14^ proposed maximizing the following partial log-likelihood to estimate $\beta$:

2 $$\begin{aligned} l(\beta ) = \sum _{i=1}^{n} \Delta _i \log \left( \frac{\exp (X_i \beta )}{\sum \nolimits _{j \in R(Y_i)} \exp (X_{j} \beta ) } \right) , \end{aligned}$$

where the risk set *R*(*t*) is the set of indices *j* for the subject whose outcome has not yet been observed at time *t* (i.e., $Y_j\ge t$).

When there are far more features than the number of observations, a reasonable assumption made in the variable selection literature is the sparsity assumption that only a small set of features are true predictors of the response, which is also adopted in this paper. The following penalized partial log-likelihood is maximized to estimate $\beta$:

3 $$\begin{aligned} pl(\beta ) = l(\beta ) - \sum \limits _{j=1}^p P_{\lambda }(|\beta _j|) , \end{aligned}$$

where the penalty term $P_{\lambda }(.)$ can represent different types of penalties (popular choices include the $L_1$ lasso penalty^20^ and the $L_1/L_2$ elastic net penalty^22^). The tuning parameter $\lambda$ controls the balance between the strength of coefficients and the penalty level and is usually chosen by cross validation. The final model consists of the features with nonzero coefficient estimates.

The above regularization methods are generally sub-optimal when *p* grows at an exponential rate of *n* (commonly seen in most genomic studies) due to algorithmic stability and accuracy. A common practice is to perform a two-stage procedure where we first reduce the dimension of the feature set by screening out unimportant features that have no marginal associations with the outcome and then apply regularization. Such methods in the first stage possess a sure screening property that the features remaining after screening still include the true set of predictors with high probability^25^. Bühlmann et al.^29^ further proposed a partial faithfulness assumption which states that a zero marginal correlation implies a zero regression coefficient in linear models to support the use of sure screening methods. In survival analysis, the principled Cox sure independence screening procedure (“PSIS”) was proposed to screen out covariates that have no association with the survival outcome in marginal Cox models^26^.

### Variable selection in the Cox model with multiple studies

Suppose we have data from *K* genomic studies where each study *k* ($1\le k \le K$) has $n_k$ observations. For study *k*, we assume the Cox model with the conditional hazard function is given by

4 $$\begin{aligned} \lambda ^{(k)}(t|X^{(k)}) = \lambda _0^{(k)}(t) \exp (X^{(k)} \beta ^{(k)}), \end{aligned}$$

where $\lambda _0^{(k)}(t)$ is the study-specific baseline hazard function, $\beta ^{(k)} = (\beta ^{(k)}_1, \ldots , \beta ^{(k)}_p)^T$ is the study-specific regression coefficients, and $X^{(k)} = (x^{(k)}_1,\ldots , x^{(k)}_p)$ is the features collected from the *k*th study. The goal of variable selection with multiple studies is to identify the active predictors $x_j^{(k)}$ with $j\in {\mathscr {M}} =\{j: \beta _j^{(k)} \ne 0 \text { for all } k \}$. Here we assume that all studies have the same sparsity pattern (i.e., $\beta _j^{(k)}=0$ for all *k* or $\beta _j^{(k)}\ne 0$ for all *k*). Under this assumption, we can accumulate potentially weak signals from each study to yield a strong evidence of active predictors. Note that because one can easily select common features across studies, we assume that *p* is common across all studies so that $x^{(k)}_j$ and $x^{(l)}_j$ correspond to the same feature *j* from the studies *k* and *l*. The extension to different *p* in different studies is straightforward and will be discussed elsewhere.

The first general framework for variable selection with multiple studies was proposed by Ma et al.^27^ where active predictors is assumed to be common to all studies, but the signal strengths (i.e., magnitudes of $|\beta _j^{(k)}|$) of those active predictors can vary among the studies. Combining marginal correlations between an outcome and each feature across studies, Ma et al. (2020) proposed the extension of sure independence screening to multiple studies in the linear models, the method known as Two-Step Aggregation Sure Independence Screening or “TSA-SIS”. With this framework of the same sparsity pattern with varied signal strength, inclusion of multiple studies yields more evidence to remove unimportant features during screening since a zero marginal correlation for any study implies a zero regression coefficient for that feature by the partial faithfulness assumption. The same framework is also adopted for the integrative analysis of multiple high-dimensional -omics data for identifying disease related biomarkers with consistent effects across multiple studies with logistic regression^30^.

Our proposed methodology is a non-trivial extension of the sure screening procedure for multiple studies of Ma et al.^27^ to the multiple Cox proportional hazards models. Unlike simpler parametric models such as linear and logistic regression models, the Cox model has the baseline hazard function as an infinite dimensional function parameter beyond the regression coefficients of interest. In linear and logistic regression, heterogeneity of multiple studies only appears as different strength of signals in $\beta ^{(k)}_j,k=1,\ldots ,K$. In our survival models, heterogeneity also comes through different study-specific baseline hazard functions $\lambda _0^{(k)}(t),k=1,\ldots ,K$, in addition to signal strength in features. Another source of heterogeneity is different censoring distributions across studies. When right censoring occurs, the exact survival time is not observed but it is only known to be larger than certain time. In the context of multiple studies, for example, one study ends in one year while another ends at five years. In this case, censored subjects in the former has survival time more than one year while time to event in the latter is only known to be larger than five years. In this paper, we address the challenging issue of additional heterogeneity in the multiple Cox models by the partial likelihood approach. To this end, we assume that the survival time and censoring time are conditionally independent given covariates. This is the key assumption that enables us to separate multiple censoring distributions from the estimation of study-specific baseline hazard functions and regression coefficients. In the medical literature, the conditional independence assumption is standard for a single study. Thus, it is reasonable to assume the same for each of multiple studies in our variable selection problem.

In this paper, we propose the Cox model based TwO-sTage variable sElection procedure for Multiple studies, namely “Cox-TOTEM”, to detect survival associated biomarkers common in multiple genomic studies. In the first stage, we extend the TSA-SIS procedure in the linear model to perform sure screening in the Cox models with multiple studies by utilizing the standardized coefficient estimates from marginal Cox models. This procedure reduces the false negative errors (i.e., missing the important features) and select the features with nonzero coefficients in all studies. In addition to the extension of the sure screening approach, we further refine the pool of features using the penalized partial likelihood in the second stage. Specifically, we propose a novel group lasso penalty in the partial log-likelihood of the Cox models to obtain the final set of features. It is natural to adopt a group lasso penalty by treating the coefficients of the same feature from multiple studies as one group. Such group lasso penalty will achieve the selection of a feature either in all studies or in none of the studies (“all-in-or-all-out”), thus identifying a common set of features across multiple studies. Below, we describe our methodology in details.

### The “Cox-TOTEM” method

**Figure 1:**
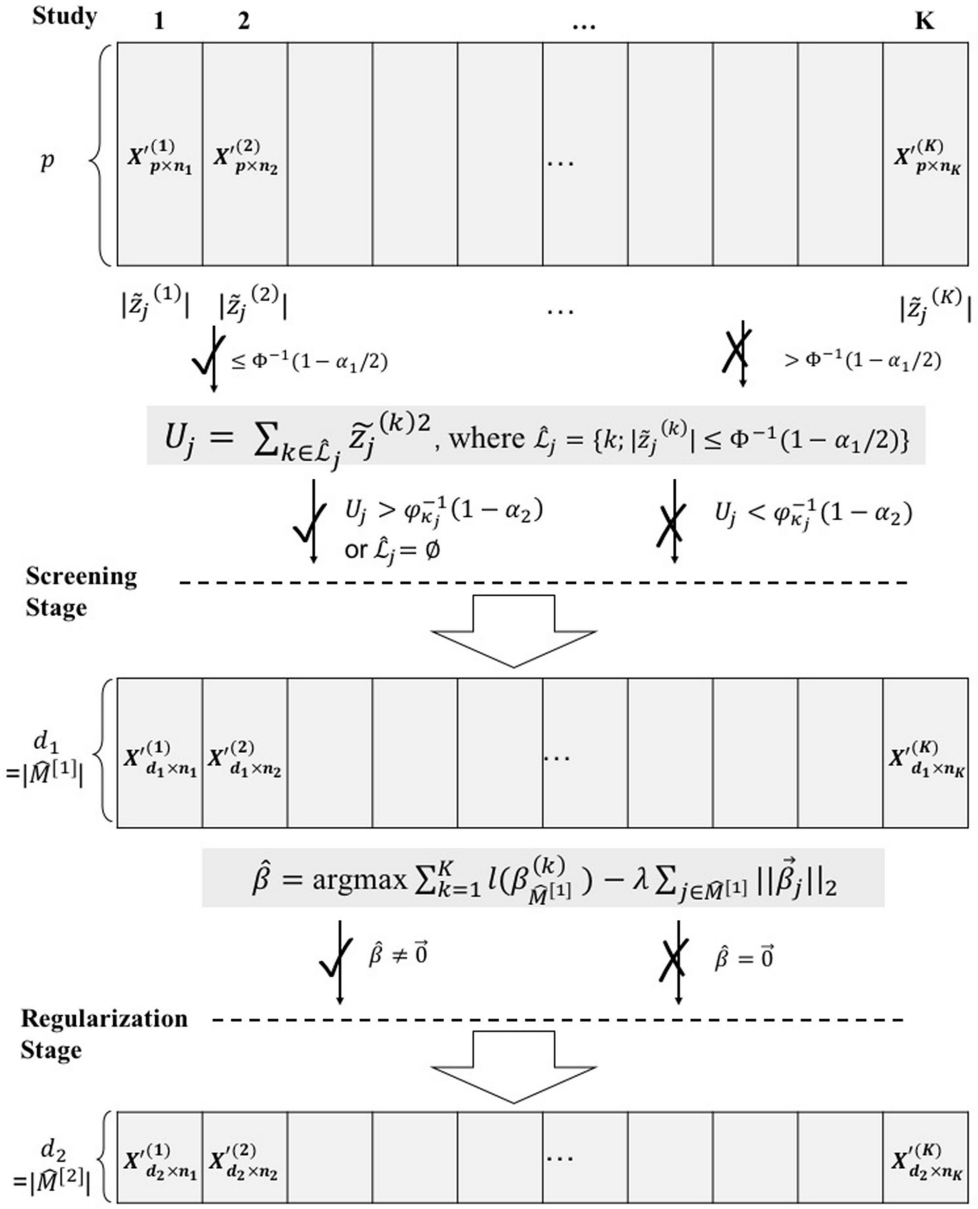
Flowchart of the proposed two-stage method Cox-TOTEM. Screening procedure applies to each feature *j* ($1\le j \le p$). “$\surd$” means passed to the next step or stage, while “*X*” means not passed to the next step or stage.

Figure 1 shows a flowchart of the proposed two-stage method Cox-TOTEM. In the first screening stage, we start by fitting a marginal Cox model with a single covariate $x_j^{(k)}$ to study *k* (i.e., $\lambda ^{(k)}(t|x_j^{(k)}) = \lambda _0^{(k)}(t) \exp (x^{(k)}_j \beta ^{(k)}_j)$). The marginal partial likelihood estimator ${\tilde{\beta }}^{(k)}_j$ for the *j*th feature in the *k*th study is a preliminary estimate used for sure screening in the first stage. Define the observed information matrix to be $I({\tilde{\beta }}^{(k)}_j)$ ($=1/ {\widehat{var}}({\tilde{\beta }}^{(k)}_j)$). The corresponding standardized marginal coefficient estimator is equal to ${\tilde{z}}^{(k)}_{j} = I({\tilde{\beta }}_j^{(k)} )^{1/2} {\tilde{\beta }}_j^{(k)}$. By the property of nonparametric maximum likelihood estimation, ${\tilde{z}}^{(k)}_{j}$ asymptotically follows the standard normal distribution when $\beta _j^{(k)}=0$. As the extension of the TSA-SIS procedure to the Cox models, we utilize $|{\tilde{z}}^{(k)}_{j}|$ to identify the studies with potential zero coefficients (i.e., weak signals or noises with small $|{\tilde{z}}^{(k)}_{j}|$) for each feature *j* in the first step:

5 $$\begin{aligned} \hat{{\mathscr {L}}}_{j} = \{k; |{\tilde{z}}^{(k)}_{j}| \le \Phi ^{-1} (1- \alpha _{1}/2) \}, \end{aligned}$$

where $\Phi$ is the CDF of the standard normal distribution. The choice of $\alpha _1$ determines the threshold to separate strong signals from weak signals. This first step does not screen out any features, but instead helps separate potential zero and nonzero coefficients for preparation of the second step.

Let ${\hat{\kappa }}_j= | \hat{{\mathscr {L}}}_{j} |$ be the cardinality of $\hat{{\mathscr {L}}}_{j}$. The second step tests whether the aggregate effect of studies with potential zero coefficients identified in the set $\hat{{\mathscr {L}}}_{j}$ is strong enough for the *j*th feature to be retained in the screening stage. Define the statistics $U_j = \sum \nolimits _{k \in \hat{{\mathscr {L}}}_{j}} {\tilde{z}}^{(k)2}_j$, which approximately follows a $\chi ^2_{{\hat{\kappa }}_j}$ distribution with degree of freedom ${\hat{\kappa }}_j$. We retain the set of features with large $U_j$ or with ${\hat{\kappa }}_j =0$:

6 $$\begin{aligned} {\hat{\mathscr {M}}}^{[1]} = \{j\in [p]; U_j > \varphi ^{-1}_{{\hat{\kappa }}_j}( 1- \alpha _2) \text { or } {\hat{\kappa }}_j = 0 \} , \end{aligned}$$

where $[p]= 1,2,\ldots ,p$ and $\varphi _{{\hat{\kappa }}_j}$ is the CDF of chi-square distribution with degree of freedom equal to ${\hat{\kappa }}_j$. The key tuning parameter $\alpha _2$ determines how many features to retain in the screening stage. The second step takes the sum of squares of ${\tilde{z}}^{(k)}_j$ from studies with potential zero coefficients as the test statistics of the *j*th feature and performs the actual screening. If the aggregate effect is strong enough, we will keep the feature and screen it out otherwise. In this way, it potentially saves those important features with weak signals in individual studies but strong aggregate effect. Define $d_1=|{\hat{\mathscr {M}}}^{[1]} |$, after the screening stage, $d_1$ features common to all studies remain and are moved on to the second stage.

Following screening, we include a group lasso penalty in the partial log-likelihoods to select the final set of features common to all studies in the regularization stage:

7 $$\begin{aligned} {\hat{\beta }}= \mathop {{\mathrm{arg\,max}}}\limits _{\beta } \sum \limits _{k=1}^K l (\beta ^{(k)}_{{\hat{\mathscr {M}}}^{[1]}}) - \lambda \sum \limits _{j \in {\hat{\mathscr {M}}}^{[1]} } ||\vec {\beta }_j ||_2, \end{aligned}$$

where $\vec {\beta }_j = (\beta ^{(1)}_j, \ldots , \beta ^{(K)}_j)$ and $\lambda$ is the tuning parameter. Note that this group lasso penalty is different from the regular group lasso penalty where the different features form pre-defined groups, e.g. multiple genes form a pathway^31^. Here we treat the coefficients of the same feature from multiple studies as one group to identify a common set of biomarkers associated with the survival. An ADMM algorithm is used to solve the above optimization problem. Detailed steps of the algorithm can be found in the Supplement. Define the set ${\hat{\mathscr {M}}}^{[2]}= \{j\in [p]; \vec {{\hat{\beta }}}_j \ne \vec {0} \}$ and $d_2=|{\hat{\mathscr {M}}}^{[2]} |$. ${\hat{\mathscr {M}}}^{[2]}$ is the final set of common features we select.

The Cox-TOTEM algorithm can be summarized as below: 
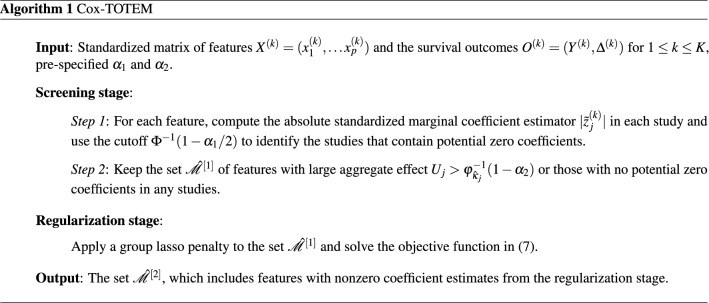


### Selection of tuning parameters

The proposed algorithm involves three tuning parameters: $\alpha _1$ and $\alpha _2$ in the screening stage and $\lambda$ in the regularization stage. Since screening is an intermediate step, the choice of $\alpha _1$ and $\alpha _2$ should be more conservative making as fewer false negative errors as possible (i.e., high sensitivity) while retaining not too many false positives (i.e., not too low specificity). A small $\alpha _1$ places stringent cutoff in selecting strong signals so more studies are included in the second step leading to more false positive errors. On the other hand, a large $\alpha _1$ tends to select very few studies causing more false negative errors. In practice, a small $\alpha _1$ is generally recommended to reduce the more serious false negative errors. The parameter $\alpha _2$ is the threshold used in the actual screening step which directly determines how many features are retained. It serves the same role as the retained model size *d* in the original sure independence screening method^25^ or the significance level $\alpha$ in the PC-simple algorithm^29^. In practice, we recommend setting $\alpha _2=0.05$, the popular significance level used in hypothesis testing. We performed a sensitivity analysis using simulation and showed that $\alpha _1=10^{-4}$ and $\alpha _2=0.05$ achieved a good balance between sensitivity and specificity in the screening stage (see Table S1 in the Supplement). When the number of features *p* becomes even larger (e.g. $p\sim 10,000$ as in our real data application) and we need to remove more features during screening, we also recommend more stringent cutoff of $\alpha _2=0.01$. Screening is by nature a fast step, we generally suggest users follow the aforementioned guideline but not use any computationally intensive data splitting or resampling procedures in choosing $\alpha _1$ and $\alpha _2$ unless otherwise specified. To determine the optimal value of $\lambda$ in the regularization stage, we propose a multi-study cross validation procedure by applying K-fold cross-validation within each study and utilizing individual survival prediction as the evaluation criteria. Details of the scheme can be found in the Supplement.

### Methods for comparison

Since no existing methods specifically look at the variable selection problem in survival model with multiple studies, we compare our method to the multiple study extension of the Cox model specific screening and regularization methods in single study. With the target index set ${\mathscr {M}}$, we screen out a feature when any study has a zero marginal coefficient for that feature. Thus, the multiple study extension of the original PSIS^26^ for sure screening in the Cox model can be regarded as MinPSIS, which ranks the features by the minimum absolute standardized marginal coefficients in all studies. Likewise, the multiple study extension of the regularization methods such as CoxLasso (lasso penalty in Cox model) or CoxNet (elastic net penalty in Cox model) take the intersection of features identified in each study, abbreviated as InterCoxLasso or InterCoxNet. In the simulation, we compare our method to a total of four methods, including two-stage methods “MinPSIS-InterCoxLasso” and “MinPSIS-InterCoxNet”, as well as direct regularization methods “InterCoxLasso” and “InterCoxNet”. For the retained model size *d* in the screening stage of other two-stage methods, we suggest more conservative cutoff of $d=n$ to mitigate the missing of true signals. For regularization methods, we use cross-validation to select the optimal tuning parameter as implemented in the R package “glmnet”^22,32^.

## Simulation

### Simulation setting

We conducted simulations to demonstrate the strength of Cox-TOTEM as compared to other methods under various scenarios.

For each study *k*, we generated $n=100$ observations, each with a survival outcome, consisting of an observed censored survival time $Y^{(k)}_i$ and a censoring indicator $\Delta ^{(k)}_i$, and a vector of $p=2000$ features $X^{(k)}_i=(x^{(k)}_{i1},\ldots ,x^{(k)}_{ip})$. $X^{(k)}_i$ was randomly sampled from a multivariate normal distribution $N_{2000}(0,\Sigma ^{(k)})$, where $\Sigma ^{(k)}_{jj}=1$ and $\Sigma ^{(k)}_{jj'}=0.5^{|j-j'|}$ for $1\le j\ne j'\le p$. The true survival time $T^{(k)}_i$ for each individual *i* was generated from the Cox model with study-specific baseline hazard function $\lambda ^{(k)}_0$ and regression coefficients $\beta ^{(k)}$ to be described below. The censoring time $C^{(k)}_i$ was generated from the exponential distribution $C^{(k)}_i\sim \exp (r^{(k)})$ with the study-specific parameter $r^{(k)}$ described below. We only assumed right censoring so the observed censored survival time $Y^{(k)}_i$ was the minimum of the true survival time and censoring time: $Y^{(k)}_i=\min (T^{(k)}_i,C^{(k)}_i)$, and the censoring indicator $\Delta ^{(k)}_i=1$ when $C^{(k)}_i> T^{(k)}_i$ and 0 otherwise. The above scheme was applied to all $K=5$ studies and the simulations were repeated for $B=100$ times.

We assumed only $s=10$ features were true predictors with nonzero coefficients and the other features had zero coefficients in all studies. The ten features with nonzero coefficients were evenly distributed among the *p* variables with equal space. We considered the following four different scenarios for a complete evaluation of our method:

- **Homogeneous strong (Homo-S)** we assumed strong signals in all studies. Let $\mu _j=-1$ for the first five features with nonzero coefficients and $\mu _j=1$ for the next five features, and let $\mu _j=0$ for the rest of features. For homogeneous study effects, we set $\beta ^{(1)}_j = \beta ^{(2)}_j = \ldots = \beta ^{(K)}_j = \mu _j$ for $1\le j\le p$. We also set the study-specific baseline hazard function $\lambda ^{(1)}_0=\lambda ^{(2)}_0=\ldots =\lambda ^{(K)}_0=1$ and $r^{(1)}=r^{(2)}=\ldots =r^{(K)}=0.2$ to be consistent across all studies.
- **Homogeneous weak (Homo-W)** the setting is the same as in Homo-S, except for now we assumed weak signals in all studies. Let $\mu _j=-0.5$ for the first five features with nonzero coefficients and $\mu _j=0.5$ for the next five features, and let $\mu _j=0$ for the rest of features.
- **Heterogeneous strong (Hetero-S)** we let $\mu _j=-1$ for the first five features with nonzero coefficients and $\mu _j=1$ for the next five features, and $\mu _j=0$ for the rest of features. To allow for between study variation in magnitudes of coefficients but not in sparsity pattern, we randomly sampled $\beta ^{(k)}_j$ from $N(\mu _j, 0.2^2)$ for $1\le k\le K$ for the features with nonzero coefficients and $\beta ^{(1)}_j = \beta ^{(2)}_j = \ldots = \beta ^{(K)}_j = \mu _j= 0$ for the other features. In addition, we also allowed for variation in baseline hazard functions and censoring distributions among studies. More specifically, we randomly drew $\lambda ^{(k)}_0$ from $\exp (1)$ and $r^{(k)}$ from $\{0.1,0.3,0.5\}$ for $1\le k\le K$ studies.
- **Heterogeneous weak (Hetero-W)** the setting is the same as in Hetero-S, except we assumed weak signals with $\mu _j=-0.5$ for the first five features with nonzero coefficients and $\mu _j=0.5$ for the next five features.

In addition to the case with $n=100$ and $p=2000$, we also conducted simulations of higher dimension with $n=200$ and $p=10,000$ and slightly weaker signals ($|\mu _j|=0.7$ for the true predictors in strong scenarios and $|\mu _j|=0.35$ in weak scenarios) and evaluated the performance under the same four scenarios. In all simulations, we used $\alpha _1=1e{-}4$ and $\alpha _2=0.05$ (and for $p=10,000$, we used $\alpha _2=0.01$) in the screening stage and chose an optimal $\lambda$ via the proposed cross-validation scheme in the regularization stage. To benchmark the performance of variable selection, we evaluated both the sensitivity and specificity as well as the average number of features selected. In addition, we also included a plot of sensitivity and specificity with varying values of tuning parameters in all methods for a fair comparison independent of the tuning parameter selection. After model selection, we fit a Cox model to all remaining features in each study and computed the sum squared errors (SSE) $\sum \nolimits _{j=1}^p\sum \nolimits _{k=1}^K ({\hat{\beta }}_j^{(k)} - \beta _j^{(k)} )^2$, where ${\hat{\beta }}_j^{(k)}$ is the Cox model coefficient estimates for *j*th feature in *k*th study, to assess the parameter estimation. A smaller SSE suggests more accurate parameter estimation.

### Simulation results

**Figure 2:**
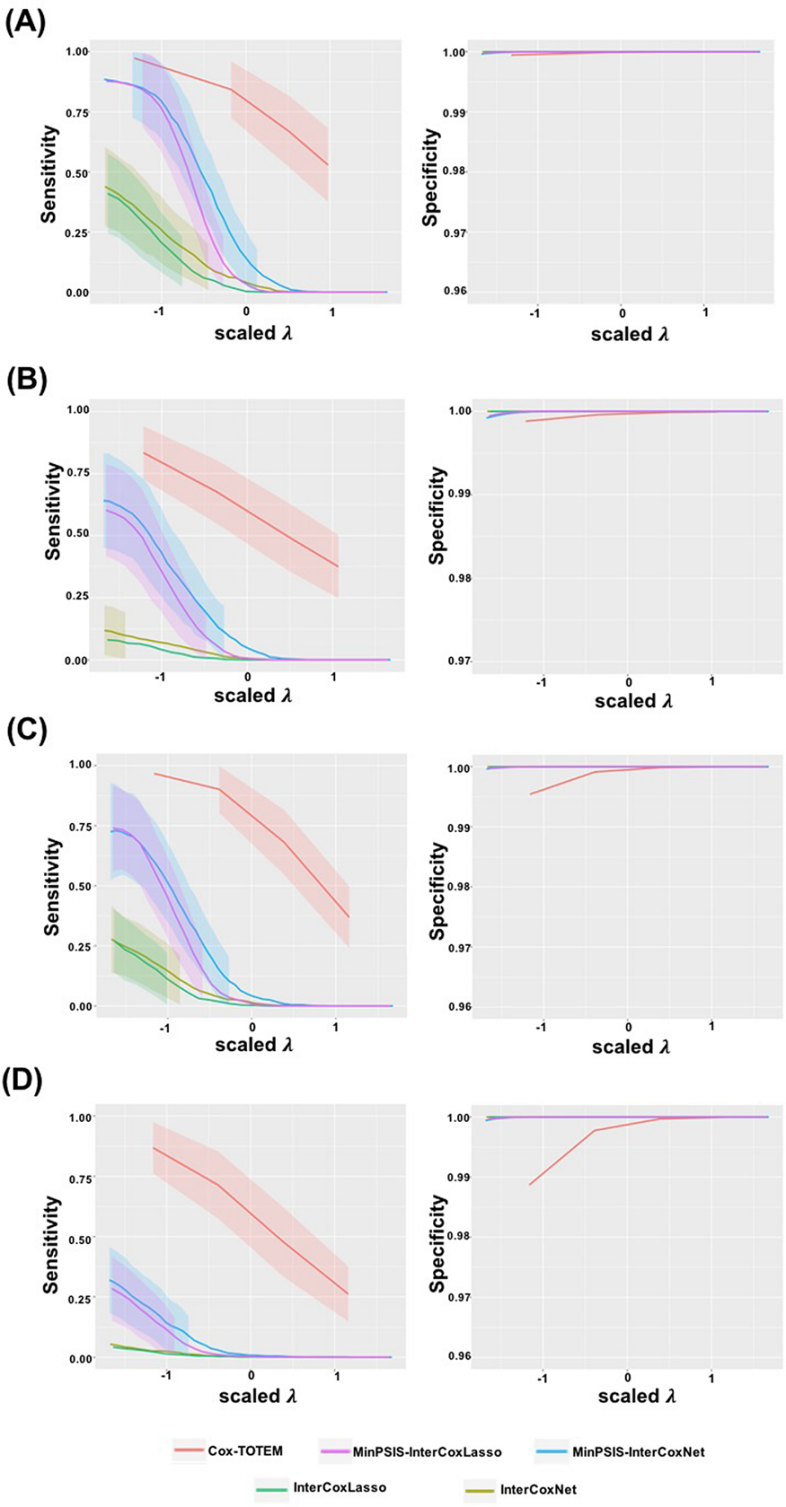
Plot of mean sensitivity (left) and mean specificity (right) against the scaled $\lambda$ values for (**A**) Homo-S; (**B**) Homo-W; (**C**) Hetero-S; (**D**) Hetero-W scenarios with $n=100$ and $p=2000$. Shades denote the standard deviations. The original $\lambda$ values are rescaled in all methods for fair comparison.

Table 1 shows the variable selection and parameter estimation performance with $n=100$ and $p=2000$. As compared to the other four methods, Cox-TOTEM greatly improves the sensitivity with almost no decrease in specificity in all four scenarios. Such gain in sensitivity is most noticeable when the signals are weak or when studies are more heterogeneous. This shows the advantage of our method in aggregating the information of multiple studies and saving those features with weak signals in one or more studies. In most cases, Cox-TOTEM retains a majority of the true signals and has more accurate parameter estimation than the other methods. Figure 2 shows the plots of mean sensitivity (left) and mean specificity (right) against the scaled $\lambda$ values for all methods in the four scenarios. Increasing $\lambda$ value decreases the sensitivity and increases the specificity for all methods. With varying $\lambda$ values, the curve of Cox-TOTEM sits above the other methods in the sensitivity plot while almost indistinguishable from the others in the specificity plot, which shows the advantage of our method regardless of the selection of the best tuning parameter. Note that in general the two-stage methods (“MinPSIS-InterCoxLasso” and “MinPSIS-InterCoxNet”) outperform the one-stage regularization methods (“InterCoxLasso” and “InterCoxNet”) as expected, encouraging the use of two-stage variable selection methods in practice. The results look very consistent in the case of $n=200$ and $p=10,000$, where Cox-TOTEM has the greatest sensitivity among all in heterogeneous and weak scenarios (see Table S2 in the Supplement).

**Table 1:** Comparison of variable selection and parameter estimation under the four different scenarios with $n=100$, $p=2000$ and $s=10$ true predictors.

Simulation scenarios	Methods	Sensitivity	Specificity	Average number of features selected	SSE
Homo-S	Cox-TOTEM	0.985 (0.004)	0.999 (0)	12.26 (0.194)	4.227 (0.269)
	MinPSIS-InterCoxLasso	0.856 (0.018)	1 (0)	9.4 (0.173)	11.36 (1.02)
	MinPSIS-InterCoxNet	0.881 (0.013)	0.997 (0.001)	14.36 (0.244)	10.79 (0.772)
	InterCoxLasso	0.37 (0.018)	1 (0)	3.7 (0.183)	37.421 (0.775)
	InterCoxNet	0.53 (0.014)	1 (0)	5.31 (0.143)	30.823 (0.684)
Homo-W	Cox-TOTEM	0.977 (0.049)	0.997 (0)	16.6 (0.385)	3.321 (0.203)
	MinPSIS-InterCoxLasso	0.464 (0.028)	0.999 (0)	5.17 (0.347)	7.646 (0.311)
	MinPSIS-InterCoxNet	0.618 (0.019)	0.997 (0)	13.07 (0.384)	6.804 (0.214)
	InterCoxLasso	0.049 (0.007)	1 (0)	0.49 (0.073)	12.032 (0.073)
	InterCoxNet	0.143 (0.011)	1 (0)	1.43 (0.11)	11.094 (0.117)
Hetero-S	Cox-TOTEM	0.968 (0.006)	0.998 (0)	13.64 (0.261)	7.969 (0.437)
	MinPSIS-InterCoxLasso	0.723 (0.023)	1 (0)	7.98 (0.226)	20.758 (1.27)
	MinPSIS-InterCoxNet	0.774 (0.015)	0.997 (0.001)	13.41 (0.26)	18.986 (0.943)
	InterCoxLasso	0.258 (0.017)	1 (0)	2.58 (0.167)	45.579 (0.716)
	InterCoxNet	0.393 (0.015)	1 (0)	3.93 (0.146)	39.842 (0.698)
Hetero-W	Cox-TOTEM	0.885 (0.01)	0.993 (0)	22.68 (0.66)	14.163 (0.163)
	MinPSIS-InterCoxLasso	0.211 (0.017)	1 (0)	2.93 (0.241)	13.118 (0.276)
	MinPSIS-InterCoxNet	0.367 (0.016)	0.997 (0)	10.01 (0.4)	11.813 (0.251)
	InterCoxLasso	0.037 (0.006)	1 (0)	0.37 (0.06)	15.738 (0.123)
	InterCoxNet	0.083 (0.008)	1 (0)	0.83 (0.075)	14.932 (0.154)

Mean results of 100 replications are reported with standard errors shown in the parentheses.

## Application to TCGA transcriptomic data

### Data description

We then applied our method to transcriptomic data from TCGA project studying the gene expression profile of four gynecological cancer types plus breast cancer (together known as Pan-Gyn cancers). Gynecologic cancers and breast cancer share a variety of generic characteristics: arising from similar embryonic origins, development all influenced by female hormone, among others^33^. Previous studies in TCGA identified shared molecular features including protein, miRNA, RNA and DNA methylation among these five cancer types^33,34^. The purpose of this application is to identify the survival associated genes common in these five cancer types, which might provide more insights into the commonalities and infer the underlying common mechanism of Pan-Gyn cancer in women.

We retrieved both the RNA-seq data and the clinical data containing survival outcomes of the five Pan-Gyn cancer types including high-grade serous ovarian cystadenocarcinoma (OV), uterine corpus endometrial carcinoma (UCEC), cervical squamous cell carcinoma and endocervical adenocarcinoma (CESC), uterine carcinosarcoma (UCS), and invasive breast carcinoma (BRCA) from TCGA data repository on Broad GDAC Firehose (https://gdac.broadinstitute.org/). Table 2 shows the total sample size and the censoring proportion of each cancer type. The censoring distributions vary greatly across the different cancer types, implying the heterogeneity in censoring patterns among studies commonly seen in real datasets. The processed RNA-seq data from TCGA include the TPM (Transcripts Per Kilobase Million) values. We first merged the five datasets by matching the gene symbols and implementing quantile normalization to remove any batch effect among the different studies. Genes with mean TPM less or equal to 5 were filtered out and 11998 genes remained. The data was also log2 transformed to be ready for the rest of the analysis.

**Table 2:** Overview of the samples from the five Pan-Gyn cancer types.

	OV	UCEC	CESC	UCS	BRCA
Sample size	304	170	301	57	981
Censoring proportion	$39\%$	$81\%$	$76\%$	$39\%$	$89\%$

### Results

**Figure 3:**
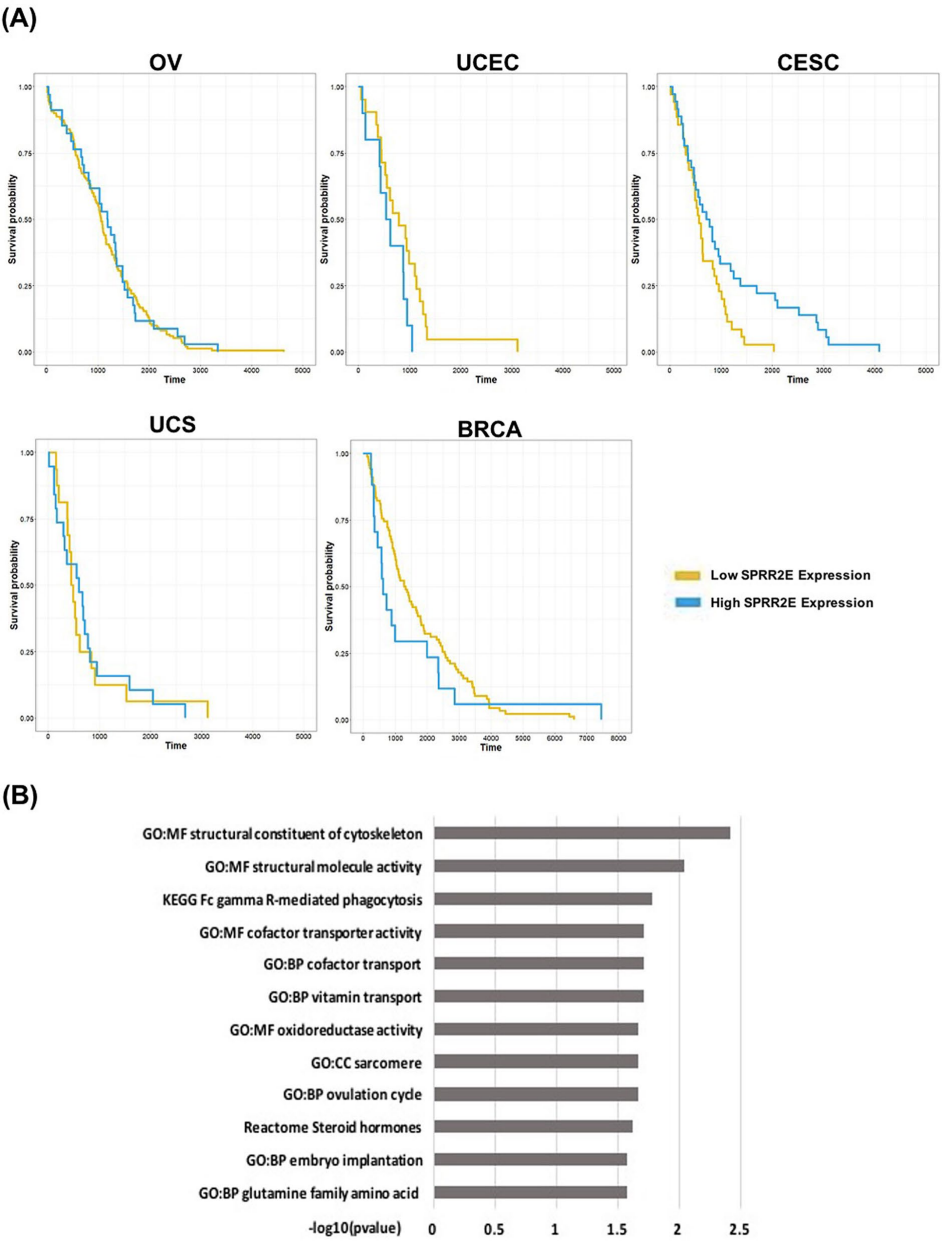
(**A**) Comparison of Kaplan–Meier curves between samples with high SPRR2E expression ($>\hbox {median}$) and those with low SPRR2E expression ($<\hbox {median}$) in the five studies. (**B**) Top pathways enriched with the 29 survival associated genes identified by Cox-TOTEM; x-axis: − log10(*p*-value), y-axis: pathway names.

We applied our method as well as the two-stage methods “MinPSIS-InterCoxLasso” and “MinPSIS-InterCoxNet” to the Pan-Gyn example. Considering the relative high dimension, we set $\alpha _2=0.01$ in the screening stage of our method and retained 268 genes after screening. The regularization stage further refined the pool to a final set of 29 genes. On the other hand, “MinPSIS-InterCoxNet” and “MinPSIS-InterCoxLasso” only detected 15 and one genes, respectively. Table 3 lists the top five genes with the largest absolute coefficient estimates from the 29 genes identified by Cox-TOTEM. None of these genes were selected by the other two competing methods. When fitting a Cox model using these genes in each cancer type, the *p*-values vary in all cancer types and are small in typically more than one cancer types. This shows the superiority of Cox-TOTEM in selecting genes associated with survival but with possibly weak signals in some studies. PPAP2C, a member of the phosphatidic acid phosphatase family, has been found to be associated with endometrioid carcinoma and ovarian cancer metastasis^35,36^. SPRR2E is part of the human epidermal differentiation complex found to be related to more than 20 different cancers including breast and endometrial cancers^37,38^. When we split the samples into a group with high SPRR2E expression ($>\hbox {median}$) and a group with low SPRR2E expression ($<\hbox {median}$) and compare their Kaplan–Meier curves in the five studies (Fig. 3A), we see a clear separation of two curves in UCEC, CESC and BRCA, but not in the other two studies, consistent with the results presented in Table 3. A complete list of the 29 genes identified by Cox-TOTEM together with their marginal Cox model results can be found in Table S3 of the Supplement.

**Table 3:** List of the top five genes from the 29 genes selected by Cox-TOTEM with the largest absolute coefficient estimates and their corresponding coefficient estimates and *p*-values (in parentheses) when fitting a Cox model in each cancer type.

	Cox model coefficient estimate (*p*-value)					MinPSIS-InterCoxLasso	MinPSIS-InterCoxNet
	OV	UCEC	CESC	UCS	BRCA		
PPAP2C	$-$ .214 (.007**)	$-$ .291 ($.131^{\cdot }$)	$-$ .130 (.244)	.155 (.455)	$-$ .074 (.594)	N.S.	N.S.
SLC19A1	.240 (.006**)	.180 (.368)	$-$ .232 (.089*)	.158 (.521)	.115 (.330)	N.S.	N.S.
SPRR2E	$-$ .058 (.619)	2.002 (.009**)	$-$ .300 (.095*)	.072 (.654)	.128 (.079*)	N.S.	N.S.
EIF4E3	$-$ .177 (.041*)	$-$ .079 (.632)	$-$ .234 ($.119^{\cdot }$)	$-$ .240 (.215)	$-$ .201 (.074*)	N.S.	N.S.
IFRD2	$-$ .133 ($.118^{\cdot }$)	$-$ .274 (.237)	.349 (.029*)	$-$ .188 (.473)	.129 (.197)	N.S.	N.S.

None of those genes were selected by MinPSIS-InterCoxLasso or MinPSIS-InterCoxNet

Significant code: $<0.01$**, $<0.1$*, $<0.15^{\cdot }$; N.S.: not selected

Since the underlying truth is unknown for real data, we also performed a pathway enrichment analysis of the 29 Pan-Gyn cancer survival associated genes identified from Cox-TOTEM using GO, KEGG^39,40^ and Reactome databases for more biological insight. Figure 3B shows the top 12 enriched pathways sorted by their corresponding − log10 *p*-values from the Fisher’s exact test. Out of these top pathways, we found pathways of important biological processes specific to female physiology such as GO:BP ovulation cycle and GO:BP embryo implantation. The enrichment in these GO pathways might reflect similar origins and development mechanism among the five Pan Gyn cancer types and validated our gene findings^33,34^.

## Discussion

In this paper, we proposed a Cox model based two-stage variable selection method called Cox-TOTEM to identify survival associated biomarkers with multiple genomic studies. In the first stage, we applied a two-step aggregation screening procedure by utilizing the standardized coefficient estimates from marginal Cox models in each study and testing whether each feature’s aggregate effect from multiple studies is strong enough to be retained. In the second stage, we started with the set of features after screening and employed a group lasso penalty to the partial log-likelihoods in Cox models to select the features simultaneously in all studies. From a meta-analytic perspective, the method improves the accuracy of variable selection in high dimension by borrowing information from multiple studies. Such advantage has also been seen in other literature when combining multiple studies for more accurate and robust results in classification and clustering^41,42^. In addition, the method is computationally favorable by greatly reducing the dimension via fast screening in the first stage and applying the efficient ADMM algorithm in the second stage. Simulations with four scenarios to describe cross-study patterns showed the method greatly improved the sensitivity especially when the signal strengths were weak or when studies were heterogeneous. A real application of the method to the RNA-seq data from TCGA identified important genes associated with survival in five Pan-Gyn cancer types. These genes were enriched in pathways specific to female physiology implying the common disease mechanism behind these cancer types.

Few methods have looked at the survival analysis problem when combining multiple high-dimensional data sets, our proposed method is one of the first to target at this significant but overlooked problem with the focus on variable selection. One important merit of our method is that we allow the studies to have different signal strengths for the same features, as well as different baseline hazard rates and censoring distributions. Such study heterogeneity is commonly seen in the biomedical literature when researchers performed survival analysis in multiple cohorts using Cox models^43,44^. In this paper, we only considered the selection of continuous covariates (e.g. gene expression) in the simulations and real data analysis, for a more general application with multi-omics data, the model can also include ordinal variables (e.g. genotype) or binary variables (e.g. DNA methylation). In case where the proportional hazards assumption does not hold in some studies, the method can be readily extended to perform variable selection in other parametric or nonparametric survival models. The performance of such extended methods needs to be further explored in both simulations and real data applications.

Other meta-analysis methods in the differential expression analysis of transcriptomic studies also consider heterogeneous sparsity pattern across studies, i.e. the set of associated biomarkers is different in different genomic studies^45^. In cases when biomarkers uniquely associated with survival in specific studies are of interest, we can include alternative regularization methods, e.g. sparse group lasso^46^, in the second stage to encourage selection of study-specific survival associated biomarkers. In addition, the directionality of effect size across studies is another major consideration in meta-analysis in practice, for example, some studies have positive coefficients thus increase the risk of failure while the others decrease the risk for the same features. Such phenomenon has also been observed in our real data example. The method can be modified to accommodate such scenarios, details of which is beyond the scope of this paper and left for future work.

The current method uses the MLE estimates of coefficients from the marginal Cox model in the screening stage and applies a group lasso penalty to the partial log-likelihoods in the regularization stage. Considering the fact that some signals may be jointly associated with the survival outcome but not marginally, we may consider using MLE in a full model with sparsity restriction to take the joint effects of features in the screening process^47^. In addition to group lasso, other types of group penalties such as group bridge or group SCAD^48^ can also be applied. An R package, CoxTOTEM, is publicly available at https://github.com/kehongjie/CoxTOTEM to implement our method.

## Supplementary Information

Supplementary Information 1.

## Data Availability

The TCGA Pan-Gyn transcriptomic and clinical data used in real data application are also publicly available at https://github.com/kehongjie/CoxTOTEM.
