# Supplementary material for: Detecting survival-associated biomarkers from heterogeneous populations
Source: Sci Rep. 2021 Feb 5;11:3203. doi: 10.1038/s41598-021-82332-y (PMC7865037; doi:10.1038/s41598-021-82332-y)
Supplement: Supplementary file 1 — Supplementary Information 1. [file 41598_2021_82332_MOESM1_ESM.pdf]

Supplementary Information for "Detecting survival-associated biomarkers from heterogeneous populations"

Takumi Saegusa, Zhiwei Zhao, Hongjie Ke, Zhenyao Ye, Zhongying Xu, Shuo Chen and Tianzhou Ma

November 17, 2020

1 ADMM algorithm for solving the optimization problem at the regularization stage

The optimization problem (7) at the regularization stage of the Cox-TOTEM algorithm is solved by the alternating direction method of multipliers (ADMM). For notational simplicity, we suppress the subscript $\hat{\mathcal{M}}^{[1]}$ in $\beta^{(k)}_{\hat{\mathcal{M}}^{[1]}}$ and use d in place of d_1. We also write $X^{(k)}$ to denote the selected features in the screening stage instead of $X^{(k)}_{\hat{\mathcal{M}}^{[1]}}$. Let $\ell_k(\beta^{(k)})$ be the partial log-likelihood function for the kth study of size n_k given by

$$\ell_k(\beta^{(k)}) = \sum_{i=1}^{n_k} \delta_i^{(k)} \log \left(\frac{\exp(X_i^{(k)}\beta^{(k)})}{\sum_{i':i' \in R(t_i^{(k)})} \exp(X_{i'}^{(k)}\beta^{(k)})} \right),$$

where $\beta^{(k)} = (\beta_1^{(k)}, \ldots, \beta_d^{(k)})^T$ is a vector of regression coefficients in the Cox model of the kth study. Let $\vec{\beta}_j = (\beta_j^{(1)}, \ldots, \beta_j^{(K)})^T \in \mathbb{R}^K$ be a vector consisting of the jth elements of $\beta^{(k)}$'s. The optimization problem (7) can be written as the following form:

$$\min_{\beta^{(k)}, k=1,\ldots,K} -\sum_{k=1}^{K} \ell_k(\beta^{(k)}) + \lambda \sum_{j=1}^{d} \|\vec{\beta}_j\|_2.$$

Let $y \in \mathbb{R}^{d\times K}$, $z \in \mathbb{R}^{d\times K}$, and $u \in \mathbb{R}^{d\times K}$. We write a row and column vector of these matrices by e.g. $y_{j\cdot} = (y_{j,1}, y_{j,2}, \ldots, y_{j,K})^T$ and $y_{\cdot,k} = (y_{1,k}, y_{2,k}, \ldots, y_{d,k})^T$. Then the equivalent optimization problem is

$$\min_{y,z} -\sum_{k=1}^{K} \ell^{(k)}(y_{\cdot k}) + \lambda \sum_{j=1}^{d} \|z_{j\cdot}\|_2, \quad \text{subject to} \quad y = z.$$

Now, the ADMM consists of the iterations

$$\begin{aligned}
y_{\cdot k}^{m+1} &= \arg\min_{y_{\cdot k}} \left\{ -\ell_k(y_{\cdot k}) + \frac{\rho}{2}\|y_{\cdot k} - z_{\cdot k}^m + u_{\cdot k}^m\|_2^2 \right\}, \quad k = 1, \ldots, K, \\
z_{j\cdot}^{m+1} &= \left(1 - \frac{\lambda}{\rho}\frac{1}{\|y_{j\cdot}^{m+1} + u_{j\cdot}^m\|_2}\right)_+ (y_{j\cdot}^{m+1} + u_{j\cdot}^m), \quad j = 1, \ldots, d, \\
u^{m+1} &= u^m + y^{m+1} - z^{m+1},
\end{aligned}$$

at the $(m+1)$th update. Here $x_+ = \max\{x, 0\}$. The update on $y_{\cdot k}$ can be carried out by the Newton-Raphson algorithm. A stopping criterion is

$$\|y^m - z^m\|_2 < \epsilon, \quad \|z^m - z^{m-1}\|_2 < \epsilon$$

for a precsribed $\epsilon > 0$.

2 Sensitivity analysis results for the choice of α_1 and α_2

Table S1: Sensitivity analysis for the choice of α_1 and α_2 using Homo-S simulation setting with $n = 100$ and $p = 2000$. All values are based on average results for 100 replications

Sensitivity/Specificity	$\alpha_2 = 0.15$	0.05	0.005
$\alpha_1 = 0.01$	0.851/0.913	0.557/0.986	0.108/0.9998
0.001	0.979/0.846	0.935/0.952	0.722/0.996
0.0001	0.999/0.838	0.991/0.943	0.936/0.993

3 Cross-validation scheme to select λ

To select the optimal tuning parameter λ in the group lasso problem, we propose a multi-study cross validation procedure. The proposed methodology is characterized by (1) k-fold cross validation within studies, and (2) prediction of survival as a performance measure. One way to perform cross validation in the analysis of multiple studies is to leave one study out as in Zhu et al. (2017). The idea behind this is that all studies are more or less comparable to each other. Our proposed group lasso, however, aims at identifying potentially different sets of predictors in each study while borrowing strength from others. Because multiple data sets exhibit selection bias and heterogeneity in patient characteristics, keeping data from all studies in cross validation serves well for the objective of the proposed group lasso.

The choice of performance measure in cross validation is closely related to the objective of statistical analysis. The existing literature van Houwelingen et al. (2006); Simon et al. (2011); Dai and Breheny (2019) utilized the variants of the partial likelihood to measure the goodness of fit through the Kullback-Leibler divergence. In this paper, we adopt the prediction of individual survival at a fixed time point. This intuitive measure is easy to interpret and simple to compute. The estimated survival probability for the ith subject in the kth study at time t in the lth cross validation is

$$\hat{S}^{(k)}_{i,CV_l}(t) = \exp\left(-e^{x_i^{(k)T}\hat{\beta}^{(k)}_{CV_l}}\hat{\Lambda}^{(k)}(t)\right),$$

where $\hat{\beta}^{(k)}_{CV_l}$ is the group lasso estimator and $\hat{\Lambda}^{(k)}(t)$ is the corresponding Breslow estimator of the cumulative hazard function. We predict a subject survived if $\hat{S}^{(k)}_{i,CV_l}(t) > 0.5$ and failure otherwise. With this performance measure, we perform multi-study cross validation as follows:

1. For each study, divide data into L pieces.

2. Leave one piece out from each study at the same time to create the training data, and then apply the group lasso algorithm.

3. Predict survival at a prescribed time t on uncensored subjects in the testing data and compare prediction with the real outcomes.

4. Repeat 2 and 3 for all L pieces.

5. Select λ that achieves most successful average prediction (i.e. maximize the prediction accuracy).

In cases where a more sparse model is favored, we will select the largest λ at which the prediction accuracy is within one standard error of the maximum one.

4 Simulation results

Table S2: Comparison of variable selection and parameter estimation under the four different scenarios with $n = 200$, $p = 10000$ and $s = 10$ true predictors. Mean results of 100 replications are reported with standard error shown in the parentheses.

Simulation scenarios	Methods	Sensitivity	Specificity	Average number	SSE
Homo-S	Cox-TOTEM	0.948 (0.01)	1(0)	11.76 (0.34)	2.47 (0.34)
	MinPSIS-InterCoxLasso	0.982 (0.005)	1(0)	10.14 (0.103)	1.119 (0.145)
	MinPSIS-InterCoxNet	0.982 (0.005)	1(0)	17.68 (0.355)	1.998 (0.142)
	InterCoxLasso	0.892 (0.016)	1(0)	8.92 (0.163)	3.705 (0.484)
	InterCoxNet	0.89 (0.014)	1(0)	8.9 (0.138)	3.779 (0.405)
Homo-W	Cox-TOTEM	0.96 (0.009)	0.999 (0)	22.26 (1.009)	2.433 (0.154)
	MinPSIS-InterCoxLasso	0.814 (0.021)	1(0)	9.82 (0.294)	1.681 (0.124)
	MinPSIS-InterCoxNet	0.814 (0.018)	0.999 (0)	22.46 (0.507)	3.068 (0.137)
	InterCoxLasso	0.102 (0.013)	1(0)	1.02 (0.126)	5.548 (0.071)
	InterCoxNet	0.2 (0.018)	1(0)	2 (0.183)	4.995 (0.103)
Hetero-S	Cox-TOTEM	0.896 (0.011)	1(0)	9.48 (0.115)	3.508 (0.278)
	MinPSIS-InterCoxLasso	0.592 (0.032)	1(0)	6.04 (0.323)	9.629 (0.736)
	MinPSIS-InterCoxNet	0.7 (0.023)	1(0)	11.68 (0.395)	8.407 (0.541)
	InterCoxLasso	0.172 (0.019)	1(0)	1.72 (0.194)	19.012 (0.436)
	InterCoxNet	0.254 (0.02)	1(0)	2.54 (0.2)	17.265 (0.442)
Hetero-W	Cox-TOTEM	0.372 (0.019)	1(0)	4.7 (0.251)	4.315 (0.129)
	MinPSIS-InterCoxLasso	0.016 (0.009)	1(0)	0.18 (0.093)	6.277 (0.062)
	MinPSIS-InterCoxNet	0.098 (0.017)	1(0)	3.6 (0.497)	6.498 (0.162)
	InterCoxLasso	0 (0)	1(0)	0 (0)	6.387 (0)
	InterCoxNet	0 (0)	1(0)	0 (0)	6.387 (0)

5 Real data results

Table S3: List of the 29 genes selected by Cox-TOTEM and their corresponding coefficient estimates and p-values (in parentheses) when fitting marginal Cox models in each cancer type.

	Marginal Cox model coefficient estimate (p-value)				
	BRCA	CESC	OV	UCEC	UCS
ARPC1B	0.19 (0.09$^{\cdot}$)	0.13 (0.34)	0.19 (0.02*)	0.5 (0.03*)	0.31 (0.11)
ASL	0.16 (0.07$^{\cdot}$)	0.47 (0.002**)	0.1 (0.2)	0.21 (0.27)	-0.05 (0.81)
CARS2	0.05 (0.62)	0.46 (0.002**)	-0.08 (0.26)	-0.47 (0.02*)	0.1 (0.58)
CCDC127	0.02 (0.83)	0.22 (0.05$^{\cdot}$)	-0.27 (0.001**)	-0.07 (0.68)	-0.29 (0.23)
CYP2R1	-0.09 (0.39)	-0.47 (0.001***)	-0.16 (0.03*)	0.07 (0.67)	-0.1 (0.53)
DES	-0.06 (0.95)	-0.4 (0.01*)	0.22 (0.004**)	0.47 (0.03*)	-0.29 (0.1)
EBNA1BP2	0.22 (0.01**)	0.4 (0.004**)	0.01 (0.89)	0.45 (0.02*)	0.03 (0.86)
EIF4E3	-0.26 (0.01**)	-0.08 (0.54)	-0.24 (0.004**)	-0.14 (0.34)	-0.22 (0.2)
FBXL18	0.12 (0.25)	0.37 (0.001**)	0.14 (0.08$^{\cdot}$)	0.12(0.59)	-0.004 (0.98)
FBXO4	-0.21 (0.06$^{\cdot}$)	0.04 (0.8)	-0.25 (0.001***)	-0.14 (0.38)	-0.09 (0.68)
GPAA1	0.33 (0.003**)	0.38 (0.002**)	-0.07 (0.34)	0.07 (0.7)	0.11 (0.51)
GPX2	-0.04 (0.68)	-0.38 (0.003**)	-0.23 (0.03*)	-0.71 (0.03*)	0.34 (0.1$^{\cdot}$)
IFRD2	0.17 (0.08$^{\cdot}$)	0.41 (0.004**)	-0.14 (0.07$^{\cdot}$)	-0.23 (0.26)	-0.24 (0.31)
IGJ	-0.07 (0.42)	-0.42 (0.001**)	0.17 (0.02*)	-0.05 (0.79)	0.01 (0.94)
KRTCAP3	-0.33 (0.001***)	0.05 (0.73)	-0.28 (0.000***)	-0.13 (0.62)	0.24 (0.24)
LOC728613	-0.14 (0.16)	0.33 (0.01**)	-0.23 (0.001**)	-0.49 (0.02*)	0.07 (0.63)
LRTOMT	-0.14 (0.21)	0.07 (0.56)	-0.2 (0.003**)	-0.43 (0.03*)	-0.02 (0.91)
MT1A	-0.13 (0.17)	0.369 (0.001**)	0.01 (0.86)	0.52 (0.02*)	0.07 (0.68)
P4HTM	-0.23 (0.07$^{\cdot}$)	0.09 (0.45)	-0.23 (0.003**)	-0.35 (0.03*)	0.09 (0.59)
PPAP2C	-0.02 (0.9)	-0.18 (0.1)	-0.26 (0.000***)	-0.24 (0.16)	0.22 (0.27)
RCL1	-0.29 (0.01*)	-0.25 (0.08$^{\cdot}$)	-0.22 (0.01*)	0.18 (0.29)	0.05 (0.77)
RIBC2	0.12 (0.22)	-0.37 (0.002**)	-0.17 (0.02*)	-0.18 (0.3)	0.18 (0.23)
SLC19A1	0.23 (0.03*)	0.41 (0.001**)	0.15 (0.07$^{\cdot}$)	0.04 (0.81)	0.19 (0.38)
SLURP1	0.17 (0.02*)	-0.36 (0.009**)	0.22 (0.004**)	-0.02 (0.92)	-0.11 (0.54)
SPRR2E	0.21 (0.001***)	-0.36 (0.01*)	-0.09 (0.42)	1.99 (0.01**)	-0.02 (0.91)
STAC2	-0.14 (0.14)	0.23 (0.04*)	0.21 (0.002**)	0.21 (0.25)	0.04 (0.83)
TREX1	-0.1 (0.37)	-0.37 (0.01**)	-0.18 (0.02*)	-0.65 (0.001**)	-0.1 (0.58)
TTC8	-0.37 (0.001**)	0.04 (0.77)	-0.05 (0.48)	-0.35 (0.07$^{\cdot}$)	-0.16 (0.46)
ZNF622	0.07 (0.4)	0.3 (0.02*)	-0.21 (0.01**)	0.32 (0.07$^{\cdot}$)	-0.09 (0.55)

Significant code: $< 0.001^{***}$,$< 0.01^{**}$, $< 0.05^{*}$, $< 0.1^{\cdot}$

References

Dai, B. and Breheny, P. (2019). Cross validation approaches for penalized cox regression. *arXiv preprint arXiv:1905.10432*.

Simon, N., Friedman, J., Hastie, T., and Tibshirani, R. (2011). Regularization paths for cox's proportional hazards model via coordinate descent. *Journal of statistical software*, 39(5):1.

van Houwelingen, H. C., Bruinsma, T., Hart, A. A., van't Veer, L. J., and Wessels, L. F. (2006). Cross-validated cox regression on microarray gene expression data. *Statistics in medicine*, 25(18):3201–3216.

Zhu, L., Ding, Y., Chen, C.-Y., Wang, L., Huo, Z., Kim, S., Sotiriou, C., Oesterreich, S., and Tseng, G. C. (2017). Metadcn: meta-analysis framework for differential co-expression network detection with an application in breast cancer. *Bioinformatics*, 33(8):1121–1129.
